# Environmental and Economic Life Cycle Assessment of Recycled Coarse Aggregates: A Portuguese Case Study

**DOI:** 10.3390/ma14185452

**Published:** 2021-09-21

**Authors:** Adriana B. Dias, João N. Pacheco, José D. Silvestre, Isabel M. Martins, Jorge de Brito

**Affiliations:** 1 c5Lab, Sustainable Construction Materials Association, 2795-242 Lisbon, Portugal; adias@c5lab.pt; 2 CERIS, c5Lab, Sustainable Construction Materials Association, 2795-242 Lisbon, Portugal; 3 CERIS, Department of Civil Engineering, Architecture and Georesources, Instituto Superior Técnico, Universidade de Lisboa, 1049-001 Lisbon, Portugal; jose.silvestre@tecnico.ulisboa.pt (J.D.S.); jb@civil.ist.utl.pt (J.d.B.); 4 Department of Materials, Laboratório Nacional de Engenharia Civil (LNEC), 1700-066 Lisbon, Portugal; imartins@lnec.pt

**Keywords:** construction and demolition waste, life cycle assessment, natural aggregates, recycled aggregates

## Abstract

The incorporation of recycled aggregates in concrete not only reduces the extraction of natural resources, but also decreases landfill disposal of construction and demolition waste. Hence, environmental impacts and costs are reduced, promoting the use of recycled aggregates and circular economy. However, the impacts of transport depend on the distance between facilities and longer distances may result in recycled aggregates being more costly and having larger environmental impact than natural aggregates. This paper discusses this topic, presents a review on the use of life cycle assessment methodology on natural and recycled aggregates for concrete, and applies this methodology in a real context pertaining the procurement of coarse aggregates to ready-mix concrete plants. A case study of two Portuguese regions, Coimbra and Lisbon, is presented. For each region, a quarry, a construction and demolition waste plant, and a ready-mix concrete plant are chosen and a comparative life cycle assessment is made. Different scenarios for the supply of natural and recycled aggregates are studied and the scenarios for recycled aggregates procurement include different hypotheses for the installation (construction and demolition waste plant or quarry) processing the construction and demolition waste into recycled aggregates. For this case study and both regions, it was found that the supply of recycled aggregates produced at the construction and demolition waste plant has lower environmental impact and cost than all other scenarios, including the provision of natural aggregates, except when it is assumed that the quarry is licensed and equipped for receiving unsorted construction and demolition waste and processing it into recycled aggregates. The paper shows that transport distance is a determining factor in the comparison of the impacts of the procurement of natural and recycled aggregates. Moreover, in the Portuguese context, the environmental impacts of the procurement of recycled aggregates may be smaller than those of natural aggregates, but cost may be larger for recycled aggregates, preventing that the most sustainable option is chosen.

## 1. Introduction

The incorporation of recycled aggregates (RAs) in concrete contributes to the European Union’s (EU) goal of 70% of non-hazardous construction and demolition waste (CDW) reuse/recycling, defined in EU Directive 2008/98/CE [1].

The technical feasibility of recycled aggregate concrete (RAC) has been demonstrated in several previous studies [2,3,4] in what concerns the mechanical and durability properties of concrete, as well as structural performance. Additionally, recent developments towards the standardization of recycled aggregate concrete (RAC) design [4] including a specific annex of prEN1992 [5] are expected to address reservations of construction agents towards RAC, contributing to its upscaling as a structural material. Moreover, under some conditions, the cost [6] and environmental impacts (EI) [6,7,8,9,10,11] associated with the procurement of aggregates by ready-mix plants are reduced when RAs are used instead of NAs.

However, this is not always the case and the EI (and cost) of RAC may be larger than those of natural aggregate concrete (NAC) [12,13,14]. Since the main motivation for RAC is the sustainability of the concrete industry, this implies that RAC may not always be the better option. Environmental and economic impacts vary from case to case and depend on transport distances, equipment, and technology used [13,15,16,17,18,19]. Due to these variations, it is important to study whether NAC or RAC is the most sustainable option on a case-by-case basis. This should take into account regional particularities, and consider the specific alternatives for the production and transport of NAs and RAs to the ready-mix plant under study.

In this context, this paper presents a comparative environmental and economic life cycle assessment (LCA) of the procurement of NAs and RAs to ready-mix plants, including their production. This LCA is made for two Portuguese regions, Lisbon and Coimbra. For each region, a quarry, a CDW plant, and a ready-mix concrete plant are selected. Three main scenarios are defined for the LCA: the production of NAs in quarries; the production of RAs in CDW plants with additional processing in quarries; and the production of RAs in quarries. The impact categories considered in the environmental LCA are global warming potential (GWP) and consumption of non-renewable energy resources (PE-NRe) due to their impact on carbon footprint and embodied energy.

## 2. Summary of Properties of Recycled Aggregates and Recycled Aggregate Concrete

NAs and RAs have different characteristics, so the incorporation of RAs results in concrete with different properties than those of NAC. RAs are less dense and more porous and deformable [20] than NAs. RAs are also weaker [21] than NAs. Therefore, typically RAC has lower compressive strength, Young’s modulus, and tensile strength than NAC, and has larger shrinkage and creep [22]. Regarding durability, Visintin et al. [23] showed a reduction of the resistance to the ingress of external agents when RAs are used. This occurs because of the large porosity of RAs and due to the typically larger water/cement ratio (to maintain workability) of RAC in comparison to NAC. However, as the strength class increases, the influence of RAs on durability tends to decrease—this occurs because high-strength concrete has a small water/cement ratio, which is characterized by small interconnectivity of pores and slow ingress of external agents into concrete [24], mitigating the detrimental influence of the porosity of RAs on durability.

In what concerns structural behavior, most resistance mechanisms rely strongly on the properties and layout of reinforcement. Therefore, the load-bearing capacity of reinforced RAC elements is not as affected by RAs as the properties of (plain) concrete [2,3]. Long- and short-term deformations increase and this is a consequence of the influence of RAs on Young’s modulus [25] and creep [26].

In order to ensure that RAC has equivalent properties to analogue NAC mixes, cement content may be increased [13]. This improves concrete properties due to a stronger, stiffer, and less porous cementitious matrix, since the same workability is met with a smaller water/cement ratio. However, increases of cement content are associated with larger cost and EI [19,27], so adoption of alternative solutions is usual.

## 3. Life-Cycle Assessment of Recycled Aggregates and Recycled Aggregate Concrete

Several studies have been carried out regarding the comparison of environmental and economic impacts of NAs and RAs and NAC and RAC. In most cases, these studies are made with specific data from producers. In the case of the comparison between NAC and RAC, some authors [7,19] resort to the same mix design for both types of concrete, while others [12,13] change mix design (e.g., increase cement ratio) when RAs are incorporated.

Marinković et al. [12] compared the EI of the production of NAC and RAC, based on local data and typical conditions in Serbia. NAC was made with fine and coarse river aggregates and RAC with natural fine river aggregates and recycled coarse aggregates. Two transport scenarios were studied, one for typical transport distances to Belgrade (100 km for river aggregates and 15 km for recycled aggregates) and the other considering the same transport distance for both types of aggregates (100 km). Four impact categories were analyzed: global warming, eutrophication, acidification, and photochemical ozone creation. For the transport scenario in typical conditions, the EI of concrete with NAs were 2% to 4% lower than those of concrete with RAs. However, when the transport distance is the same, the EI of NAs are 11% to 37% smaller than those of RAs, depending on the impact category, due to the transport type (RAs are transported by trucks while NAs are transported by ships). As shown in this study, the transport mode and distance may result in RAs with larger environmental impact than NAs.

Tošić et al. [7] studied different aggregate types and transport scenarios to determine the optimal solution for concrete production with the lowest environmental and economic impacts. An LCA combined with multi-criteria optimization was used. Four types of ready-mix concrete were considered, with fine and coarse river aggregates, fine and coarse crushed stone, and coarse recycled concrete aggregates, all produced in Serbia. The alternatives were evaluated according to the energy use, the environmental load, the waste generation, the mineral resource depletion, and an economic criterion. In economic terms, the solution with river aggregates has the lowest impacts and, in environmental terms, the solution with 100% of coarse recycled aggregates is the best alternative. To overcome the problem of natural resource depletion in Serbia, the increase of the cost of NA extraction was proposed.

Hossain et al. [8] applied LCA to assess and compare the EI of the production of fine and coarse NAs and RAs using data from producers in Hong Kong. The results showed a reduction of 65% in greenhouse gas emissions and 58% in non-renewable energy consumption in the production of recycled coarse aggregates when compared to NAs. The overall EI in the production of RAs can be reduced up to 50% when compared to the production of crushed stone. Additionally, the use of RAs leads to a reduction of landfill disposals and mitigates the shortage of NAs.

Braga et al. [6] presented a comparison of the life cycle impacts of concrete with NAs and RAs using data collected from Portuguese companies. In comparison to granite, limestone has smaller environmental impact, while the natural fine aggregate with lower impact is river sand (in comparison to crushed fine aggregates). When RAs are used, the EI and costs associated with the production and transport are significantly reduced. However, the use of RAs is often associated with the increase of the concrete cement content and this leads to higher EI when the LCA concerns concrete rather than coarse aggregates. Regarding the economic impacts, the use of limestone instead of granite leads to 50% savings. When granite and RAs are compared, RA results in cost savings of 80%.

Fraj and Idir [13] compared the environmental impact of RAC with that of NAC. Data were collected from companies located in Paris. It was found that the results are very dependent on the transport distance and on the amount of RAs incorporated (because this study assumed that additional cement was added when RAs were used to offset the decrease of compressive strength due to the RAs). In this study, the impacts of the production of RAs are generally higher than those of NAs, namely in energy consumption (19% higher) and global warming (34% higher) impact categories. However, regarding the influence of transport distance, NAs were found to be more advantageous in terms of GWP when the quarry is located at a maximum distance of 22 km.

Estanqueiro et al. [15] presented a comparison of the use of NAs and RAs in concrete, through an environmental LCA, using site-specific data supplied by Portuguese companies. Three scenarios were considered, one with NAs and the other two with RAs, one using a fixed recycling plant and the other using a mobile recycling plant. When compared to NAs, the use of RAs only presents better environmental performance in terms of land use and respiratory inorganics impact categories. However, using fine recycled aggregates instead of sending them to a landfill reduces EI by 23% in terms of GWP, when compared to NAs. It was also shown that the location of the quarry, demolition site, and concrete plant have a large influence on the comparison between NAs and RAs.

Park et al. [14] analyzed the impacts of dry and wet methods for removal of attached mortar from recycled concrete aggregates during their production using the Life Cycle Index Database of Korea. In the dry method, the attached mortar of this type of RA is separated from the aggregate surface by using two cone crushers, while in the wet method a complex system that includes a jaw crusher, two cone crushers, and air blowers is used. The impacts of wet production were up to 16% and 40% higher than those of dry production, regarding eutrophication and acidification potential, respectively. However, the wet production was more effective, with better quality aggregates. It was also found that the impacts of RA production were up to twice as high as those of NAs (gravel from sea, land, and mountains) due to the amount of energy required for their production. However, in terms of abiotic resource depletion potential, the results were higher for NAs because of the use of natural resources, since RAs result from construction waste.

**Figure 1:**
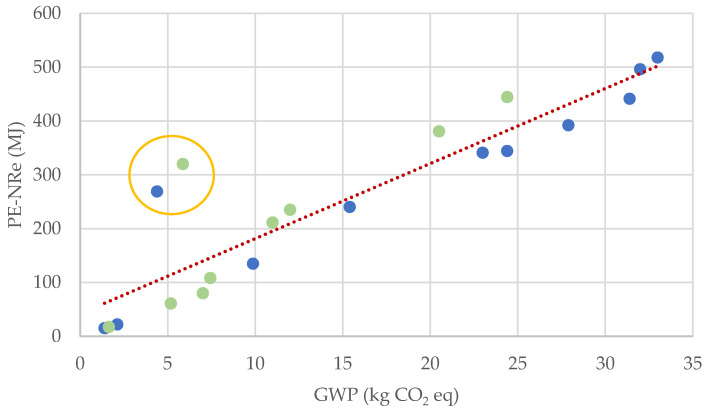
GWP vs. Pe-NRe, per tonne of aggregate. Data from the EI appraised in this literature review (blue for NA and green for RA).

Figure 1 shows the EI of the production and transport stages of the NAs and RAs of the studies described above in terms of GWP and PE-NRe. The results in blue are related to NAs and the ones in green to RAs. The results follow a linear relationship, with an R^2^ value (coefficient of determination which, in this case, gives the proportion of the variation in the value of PE-NRe that is predictable from the change in the value of GWP) very close to 1 (0.97) when the outliers circled in orange, away from the trend line, are neglected. When these results are included, the R^2^ value decreases to 0.83.

Other authors have studied the environmental and economic impacts of aggregates, considering other types of data. Some of their results are presented below.

Jullien et al. [16] analyzed the energy consumption and atmospheric emissions of the production of 1 tonne of fine and coarse natural aggregates in three different quarries in France. The results were compared by seven indicators of the LCA methodology. It was found that the energy consumption varies from site to site, which is reflected in different EI. Some differences between facilities, related to processes and equipment, explain the differences on the results. Fine aggregates have large impacts in terms of energy consumption due to the onsite production process. It was also found that the use of explosives has an impact of less than 1% on the total impacts.

Simion et al. [9] quantified the impacts of the production of RAs from CDW and compared them with those of the production of NAs (of undisclosed lithology), using LCA. The analysis was carried out in the Italian Emilia Romagna region and was based on primary data collection. The results showed that the EI of the production of RAs are about 40% lower than those of NAs production, where the GWP for RAs is 7 times less than for NAs.

Faleschini et al. [28] also studied the EI of the NA and RA production in the Italian context. The case study was an integrated plant for the extraction of natural resources and recycling of CDW, using site-specific data. It was found that the integration of the processing of NAs and RAs on the same plant leads to a reduction of the EI of production. To reduce the consumption of non-renewable energy, a photovoltaic installation was assumed in a sensitivity analysis. This option allowed increasing the delivery distance by 45 km for the global warming potential category of the NAs and RAs production chains, comparing with the solution of the integrated plant without photovoltaic installation.

Rosado et al. [10] compared through LCA, with site-specific data, the production of basalt NAs with that of RAs produced from mixed CDW. These aggregates are meant for road construction in Southeast Brazil. It was found that, for global warming and non-renewable energy impact categories, RAs are preferable to NAs. Moreover, if the distance from the recycling facility to the consumer is below 20 km, the production of RAs results in smaller EI for all categories.

The LCA of the production of NAs and RAs includes all stages regarding extraction and processing of the aggregates to be used in concrete, while the LCA of concrete with NAs and RAs appraises the production and transport to the ready-mixed plant of the various constituents of the concrete, namely cement, admixtures, water, and aggregates (natural or recycled), and the production of concrete itself.

Kurda et al. [11] compared the EI of different concrete mixes produced in Portugal. The mixes had varying incorporation ratios of fly ash (FA) and fine and coarse recycled concrete aggregates. It was found that the increase of fine recycled concrete aggregates does not change the abiotic depletion potential (ADP), but the incorporation of FA has a positive influence on this impact category. Regarding GWP, this category also decreases when FA is incorporated to replace cement. The results were practically the same when fine RAs were used instead of fine NAs since the decrease in GWP due to the smaller transport distance of the fine RAs is offset by the smaller GWP of the production of fine NAs (river sand, which is not crushed). Regarding coarse aggregates, the impacts of RAs were lower due to the production and transport stages (production of NAs results in larger GWP than that of RAs since coarse NAs are crushed limestone).

Göswein et al. [17] focused on the transportation impacts related to the environmental assessment of concrete mixes with incorporation of FA and RAs. Based on two Portuguese cities as case studies, a method combining LCA and geospatial analysis was proposed. Transportation was found to be relevant especially when the mixes have high incorporations of either FA or RAs, due to the transport distance of both materials from suppliers to concrete plants.

Colangelo et al. [18] analyzed the environmental impact of RAC in a specific region of Southern Italy. Through LCA, a comparison between several concrete mixes with different incorporations of CDW, marble sludge, and cement kiln dust was made. Several disposal/recovery scenarios were considered, taking into account the amount of aggregates disposed of in landfills, the amount of recycling, and the distance from the site. The EI increase when distance increases and the smaller impacts are related with mixes with CDW, while NAC is the mix with the highest EI.

Pradhan et al. [19] compared the EI of the production of NAC and RAC. Data related to the production of aggregates were collected from facilities in India. It was found that higher EI are mostly caused by the cement content, followed by transportation activities.

As understood from this appraisal, in general RA and RAC have smaller environmental impacts than NAC. However, differences related to the shape of aggregates (e.g., the use of river or crushed gravel), to the mix design (e.g., increases of cement content of RAC when RAs are used), to processing [16], and transport [17] lead different authors to reach distinct findings. Moreover, these studies are accounting for the impacts associated with the production of aggregates and concrete (including transport). When RAs are used, since landfill disposals are reduced and the extraction of NA is prevented, there are social and uncountable environmental benefits that are not being considered. This is a typical limitation of LCA on RA and RAC that could be accounted for with other methods, such as multi-criteria methods [29] or using consequential instead of attributional LCA. Such methods are outside the scope of this paper.

## 4. Case Study in the Portuguese Context

### 4.1. Environmental Life Cycle Assessment

In this study, an LCA is carried out to compare the environmental and economic impacts of NAs and RAs. This requires the definition of functional unit, system boundaries, and life cycle inventory. The functional unit was chosen as 1 tonne of aggregates and implies that the impacts of NAs and RAs will be compared for the same mass of aggregate. The system boundaries define which processes are included (in this case production and transport, including the production and delivery of raw materials, the production of the coarse aggregate, and its transport to the ready-mix plant) and the life cycle inventory gives information about the data collection.

For each of the two regions analyzed (Lisbon and Coimbra), different industrial units were selected:

- A quarry for the extraction of NAs;
- A demolition site for the CDW collection;
- A CDW plant for the reception of these wastes and production of RAs;
- A ready-mix concrete plant, where concrete is produced and raw materials (including coarse aggregates) received.

The ready-mixed plants selected for this study are those of an ongoing research project that aims at the industrial production of recycled aggregate concrete at those plants. The quarry selected for each region is the one that usually supplies NAs to each ready-mix plant. The CDW plants were selected based on preferences of the owners of the ready-mix plants.

**Figure 2:**
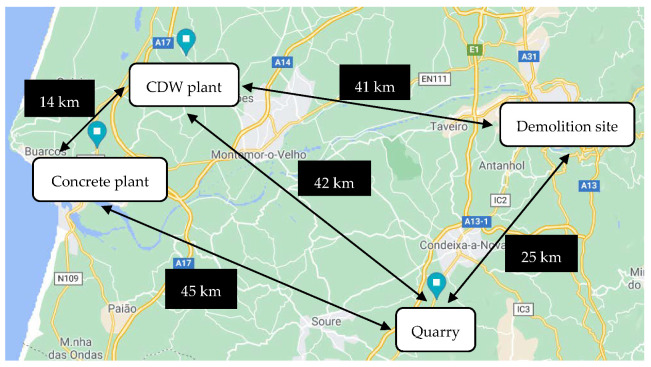
Transport distances of the aggregates through the main operating locations in Coimbra region.

**Figure 3:**
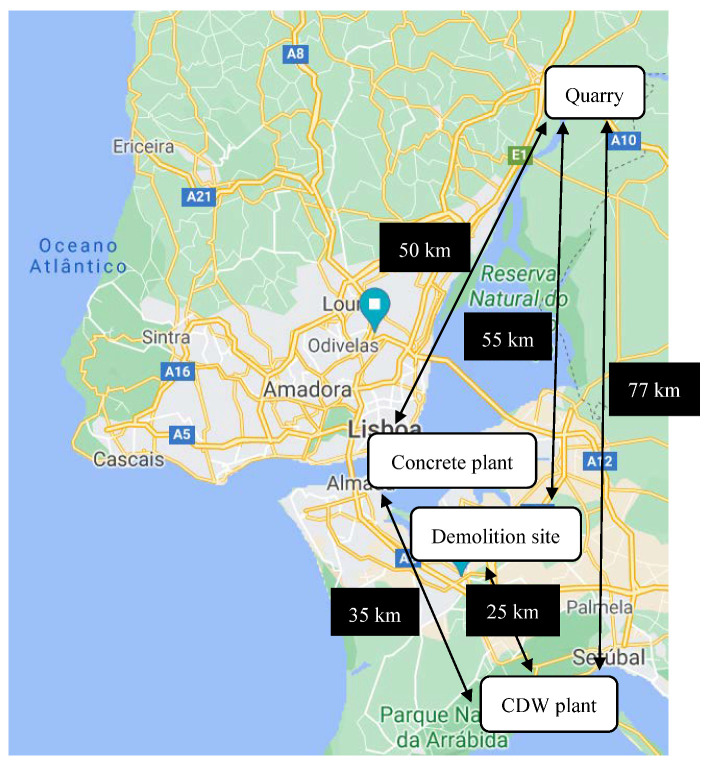
Transport distances of the aggregates through the main operating locations in Lisbon region.

Transport distances between the main operating locations of Coimbra are presented in Figure 2 and of Lisbon in Figure 3.

In Table 1, a comparison of all transport distances between the two regions is shown. The main differences between the two regions are the distance from the demolition site to the CDW plant, which is about 62% longer for the region of Coimbra, and the distance from the CDW plant to the ready-mix concrete plant, which is 40% shorter for the region of Coimbra. These differences have opposite effects: increased distances from demolition site to CDW are associated with larger impacts from the procurement of RAs, while decreases in distances from CDW plant to ready-mix concrete plant result in smaller impacts for the same raw material.

LCA was based on different scenarios shown in Table 2, which are explained below:

- Scenario 1—NAs are produced at the quarry, where they are crushed, washed, and sieved prior to their delivery to the ready-mix concrete plant;
- Scenario 2a—CDW is transported from the demolition site to the CDW plant, where the CDW is sorted, crushed into RAs, and sieved, and then the RAs are delivered to the ready-mix concrete plant;
- Scenario 2b—CDW is transported from the demolition site to the CDW plant, where the CDW is sorted, crushed into RAs, and sieved, and then the RAs are transported to the quarry, where they are washed and sieved, and finally RAs are delivered to the ready-mix concrete plant;
- Scenario 2c—CDW is transported from the demolition site to the CDW plant, where the CDW is sorted (therefore, contaminants are minimized), then the CDW is transported to the quarry, where it is crushed, washed, and sieved, and finally RAs are delivered to the ready-mix concrete plant;
- Scenario 3—CDW is transported from the demolition site to the quarry, where it is received in conformity with a suitable code of the European List of Wastes; then, the CDW is sorted and the content of contaminants is reduced. Afterwards, CDW is crushed into RAs, which are washed and sieved. Finally, RAs are delivered to the ready-mix concrete plant.

The life cycle inventory data, collected from previous studies from Portuguese companies, are shown in Table 3. The values correspond to the production and transport processes of NAs and RAs.

**Figure 4:**
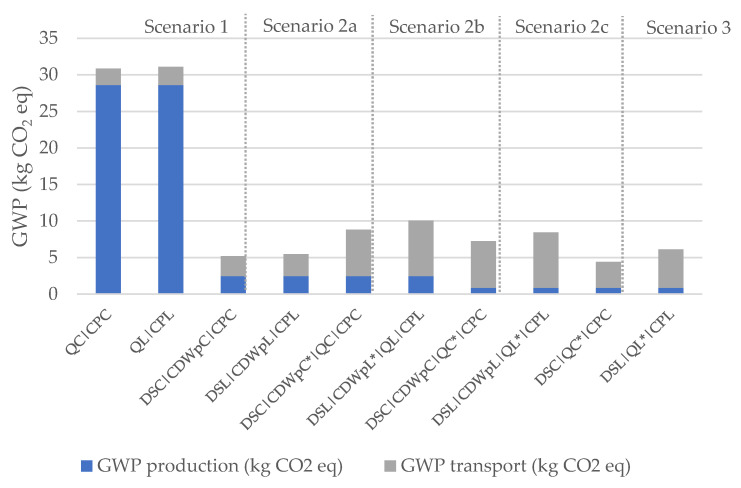
GWP for production and transport of 1 tonne of aggregates.

**Figure 5:**
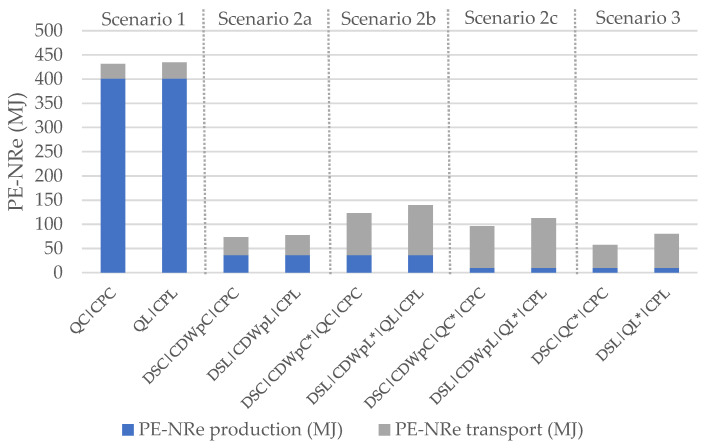
PE-NRe for production and transport of 1 tonne of aggregates.

The results in terms of EI of the production and transport are presented below. Table 4 and Figure 4 show the results in terms of GWP and Table 5 and Figure 5 in terms of PE-NRe. In these tables and figures:

- QC is the quarry of Coimbra, while QL is the quarry of Lisbon;
- CPC and CPL are the ready-mix concrete plants of Coimbra and Lisbon;
- DSC and DSL are the demolition sites of Coimbra and Lisbon;
- CDWpC is the CDW plant of Coimbra and CDWpL is the CDW plant of Lisbon;
- Places marked with * represent the locations where crushing takes place, differentiating crushing at CDW plant (scenario 2b) from crushing at the quarry (scenario 3).

In Table 4, the best results are marked in green and the worst in orange. In terms of GWP and PE-NRe:

- The impacts are the lowest for the region of Coimbra in Scenario 3 and for the region of Lisbon in Scenario 2a, and the highest for the first scenario in both regions;
- Regarding production impacts, the best results are related to the production of RA directly at the quarry (Scenarios 2c and 3);
- Regarding the impacts of transport, the best results are those of Scenario 1, because of the reduced transport distances between facilities. However, differences between Scenario 1 and 2a are small in both regions;
- Concerning RAs, the best solution for immediate implementation is Scenario 2a (procurement of RAs produced and delivered by the CDW plant) for both regions. Moreover, this solution results in much smaller impacts than the procurement of NAs.

In this preliminary study, the functional unit was defined in terms of mass. Since RAs are less dense than NAs, the required mass of RAs to replace NAs in the production of concrete is lower and the functional unit is not exactly the same. However, as seen in Section 2, this is the typical option of comparative LCA concerning the production of NAs and RAs.

### 4.2. Economic Life Cycle Assessment

Production costs were collected from a previous study related to Portuguese companies [31]. For NAs, the production cost assumed was 4.60 €/t, while the cost for the production of RAs was assumed as 2.00 €/t. In what concerns transportation, the average transport cost in Europe in 2016, in an articulated lorry with a maximum capacity of 27 tonnes was assumed. This cost is 4.88 × 10^−2^ €/t·km [32].

**Figure 6:**
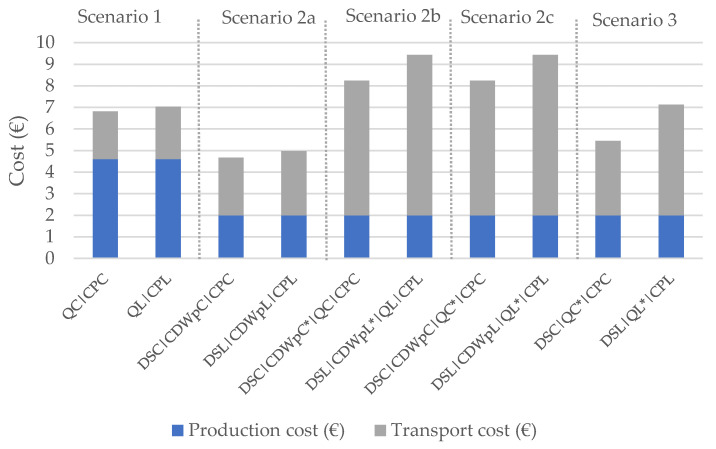
Cost for production and transport of 1 tonne of aggregates.

The economic impacts of the production and transport are shown in Table 6 and Figure 6. It is shown that:

- Scenario 2a is the least costly and the one with the most feasible and immediate implementation because there is no need to further investment on equipment or in licenses to receive CDW at the quarry;
- Scenarios 2b and 2c present the highest costs;
- In terms of production cost, the results are better for the scenarios with RAs;
- On the other hand, transport costs are lower for the first scenario, concerning NAs, due to the shorter transport distance.

Regarding costs, it is highlighted that:

- The same cost was considered for RAs produced either at the CDW plant or at the quarry;
- The investment needed for the quarry to be able to produce RAs with CE marking was not accounted for (Scenarios 2c and 3);
- The licensing cost for the quarries to be able to receive CDW was also not considered (Scenarios 2c and 3).

### 4.3. Discussion of Results and Sensitivity Analyses

As understood in Section 4.1 and Section 4.2, Scenario 2a is the best option in what concerns both environmental and economic impacts. Moreover, this scenario has the advantage of not requiring investment and licensing of quarries in order to receive and process CDW to RAs.

Based on the impacts per km of transport, the distance for which environmental and economic impacts of Scenario 1 (production and delivery of NAs from the quarry to the ready-mix plant) are the same as those of Scenario 2a (production and delivery of RAs from the CDW plant to the ready-mix plant) was calculated. It was found that:

- Scenario 2a is only less costly than Scenario 1 when the transport distance of RAs is 53 km shorter than the transport distance of NA.
- In terms of GWP and PE-NRe, Scenario 2a is better than Scenario 1 when RAs are less than 500 km more distant from the ready-mixed plant than NAs, which will occur virtually always in the Portuguese context.

**Figure 7:**
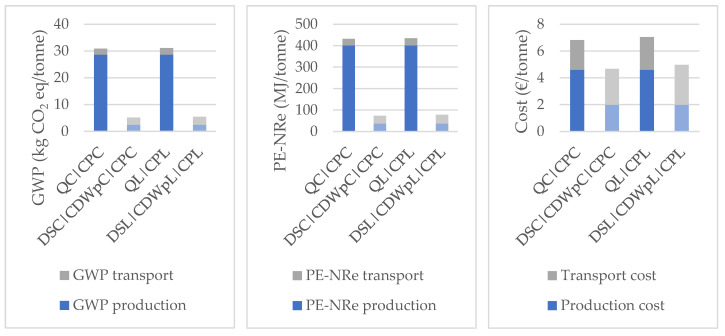
Comparison between Scenario 1, darker color, and Scenario 2a, lighter color.

In order to better understand the differences between production and transport for both scenarios, Figure 7 shows a comparison between them. The main difference is related to the EI of the production of NAs, which are about 90% higher than the impacts of the production of RAs. This occurs due to the activities related to the extraction of NAs at the quarries. Regarding economic impacts, the difference is less significant, with a reduction of about 30% when replacing NAs with RAs. This leads to two major conclusions: (i) for the modelling of costs and impacts used in this paper (representative of the Portuguese context), concrete plants may opt for NAs due to the cost in cases in which RAs would be the most environmentally friendly option; (ii) for the same transport distance, the procurement of RAs has smaller environmental impacts than that of NAs.

**Figure 8:**
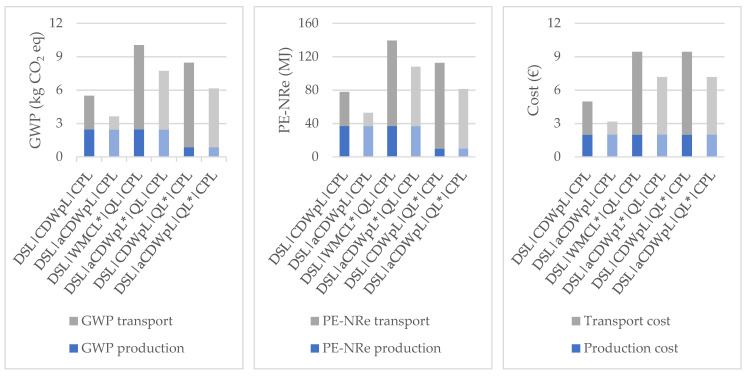
Original CDW plant (CDWp), darker color and alternative CDW plant (aCDWp), lighter color.

Since the differences between the impacts of NAs and RAs could be larger if the CDW plant were closer to the ready-mix plant, an analysis considering an alternative CDW plant in Lisbon was carried out. The alternative CDW plant was selected so that it would be simultaneously closer to the demolition site, to the quarry, and to the ready-mix concrete plant. The differences of the distances between the first selected CDW plant and the alternative one are shown in Table 7. Figure 8 compares the environmental and economic impacts when either of the CDW plants of Lisbon are considered. With the alternative plant, the impacts of transport were reduced by about 28% in terms of GWP and costs and about 27% in terms of PE-NRe.

## 5. Conclusions

The replacement of NAs with RAs is seen as an environmental-friendly option that reduces the EI associated to the extraction of coarse natural aggregates. Therefore, the environmental impact of ready-mix concrete production also decreases. However, since EI of high bulk materials (such as aggregates for construction) are strongly dependent on transport distance, the procurement of RAs may actually result in larger EI than the supply of NAs. Therefore, this analysis should be made on a case-to-case basis. Economic impacts should also be assessed, since RAs must be cost-competitive in order to be a sustainable solution.

In this study a review of the state of the art of the use of NAs and RAs in concrete is made, comparing the environmental and economic impacts of both types of aggregates. Additionally, an environmental and economic LCA was carried out considering two Portuguese regions, Coimbra and Lisbon. For each region, a quarry, a CDW plant, and a ready-mix concrete plant were selected. Three main scenarios were defined, in order to evaluate the impacts of the several solutions.

Regarding the EI, the findings are very similar for the two environmental impact categories analyzed (global warming potential and consumption of non-renewable primary energy). The lowest EI are related to Scenario 3, for the region of Coimbra, in which the CDW is directly sent from the construction site to the quarry, sorted, and processed into RAs, which are then delivered to the ready-mix concrete plant. However, the immediate implementation of this scenario is impaired due to the need to invest in equipment and personnel at the quarry and, foremost, legal restrictions for the reception and processing of waste at quarries. For the region of Lisbon, the best Scenario is 2a, in which the CDW is transported from the demolition site to the CDW plant and, after processing, RAs are delivered to the ready-mix concrete plant.

Regarding economic impacts, the best option and the one that could be immediately implemented is Scenario 2a, for both regions.

The worst EI are related to the first scenario, representing the current situation, in which NAs are produced at the quarry and then are delivered to the ready-mix concrete plant. The impacts of production are always lower for the scenarios with RAs but the impacts of transport are higher due to increased total travel distances between facilities and demolition site (NAs only travel from quarry to ready-mix plant).

The worst economic impacts are related to the scenarios in which CDW is processed both at CDW plant and at the quarry. This is a consequence of the impacts of transport, due to the increase of travel distance.

In order to study the influence of transport distance, an alternative CDW plant for the region of Lisbon was studied in a sensitivity analysis. This plant is closer to the demolition site, quarry, and ready-mix concrete plant. This solution resulted in a reduction of about 28% in terms of environmental and economic impacts.

Future studies will focus on the comparison of the EI and cost associated with the production of natural and recycled aggregate concrete. Some aspects related to concrete mix design that were not considered in this paper will be taken into account, such as the fact that the density of recycled aggregates is lower than that of natural ones (therefore, 1 tonne of natural aggregates will be replaced with less than 1 tonne of recycled aggregates) and the consideration of probable changes in mix design, needed to offset the influence of RAs on concrete properties, in the LCA.

## Figures and Tables

**Table 1:** Transport distances (in km) of the aggregates through the main operating locations.

Region	Location	CDW Plant	Quarry	Ready-Mix Concrete Plant
Coimbra	Demolition site	40.7	25.3	-
	CDW plant	-	41.6	14.1
	Quarry	-	-	45.5
Lisbon	Demolition site	25.4	55.1	-
	CDW plant	-	76.9	35.5
	Quarry	-	-	50

**Table 2:** Transport of the aggregates and main operations for each scenario.

**Current scenario** (**1**)					Quarry (NA crushing)	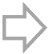	Concrete plant
**Intermediate scenario** (**2a**)	Demolition site	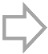	CDW plant (RA crushing)		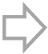		Concrete plant
**Intermediate scenario** (**2b**)	Demolition site	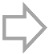	CDW plant (RA crushing)	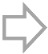	Quarry	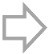	Concrete plant
**Intermediate scenario** (**2c**)	Demolition site	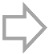	CDW plant	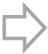	Quarry (RA crushing)	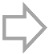	Concrete plant
**Future scenario** (**3**)	Demolition site		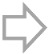		Quarry (RA crushing)	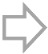	Concrete plant

**Table 3:** EI for the production and transport of NAs and RAs.

	Production of 1 Tonne of RAs at the Quarry [30]	Production of 1 Tonne of NAs [31]	Production of 1 Tonne of RAs [31]	Transport by Truck (1 Tonne·km) [31]
**GWP** (kgCO_2_eq)	8.72 × 10^−1^	2.86 × 10^1^	2.45 × 10^0^	4.98 × 10^−2^
**PE-NRe** (MJ)	9.98 × 10^0^	4.01 × 10^2^	3.86 × 10^1^	6.73 × 10^−1^

**Table 4:** GWP for production and transport of 1 tonne of aggregates.

Scenarios		GWP Production (kg CO_2_ eq)	GWP Transport (kg CO_2_ eq)	GWP Total (kg CO_2_ eq)
1	QC|CPC	28.6	2.3	**30.9**
1	QL|CPL	28.6	2.6	**31.1**
2a	DSC|CDWpC|CPC	2.5	2.7	**5.2**
2a	DSL|CDWpL|CPL	2.5	3.0	**5.5**
2b	DSC|CDWpC *|QC|CPC	2.5	6.4	**8.8**
2b	DSL|CDWpL *|QL|CPL	2.5	7.6	**10.0**
2c	DSC|CDWpC|QC *|CPC	0.9	6.4	**7.2**
2c	DSL|CDWpL|QL *|CPL	0.9	7.6	**8.5**
3	DSC|QC *|CPC	0.9	3.5	**4.4**
3	DSL|QL *|CPL	0.9	5.2	**6.1**

* Locations where crushing takes place.

**Table 5:** PE-NRe for production and transport of 1 tonne of aggregates.

Scenarios		PE-NRe Production (MJ)	PE-NRe Transport (MJ)	PE-NRe Total (MJ)
1	QC|CPC	401.0	30.6	**431.6**
1	QL|CPL	401.0	33.7	**434.7**
2a	DSC|CDWpC|CPC	36.8	36.9	**73.7**
2a	DSL|CDWpL|CPL	36.8	41.0	**77.8**
2b	DSC|CDWpC *|QC|CPC	36.8	86.0	**122.8**
2b	DSL|CDWpL *|QL|CPL	36.8	102.5	**139.3**
2c	DSC|CDWpC|QC *|CPC	10.0	86.0	**96.0**
2c	DSL|CDWpL|QL *|CPL	10.0	102.5	**112.5**
3	DSC|QC *|CPC	10.0	47.7	**57.6**
3	DSL|QL *|CPL	10.0	70.7	**80.7**

* Locations where crushing takes place.

**Table 6:** Cost for production and transport of 1 tonne of aggregates.

Scenarios		Production Cost (€)	Transport Cost (€)	Total Cost (€)
1	QC|CPC	4.6	2.2	**6.8**
1	QL|CPL	4.6	2.4	**7.0**
2a	DSC|CDWpC|CPC	2.0	2.7	**4.7**
2a	DSL|CDWpL|CPL	2.0	3.0	**5.0**
2b	DSC|CDWpC *|QC|CPC	2.0	6.2	**8.2**
2b	DSL|CDWpL *|QL|CPL	2.0	7.4	**9.4**
2c	DSC|CDWpC|QC *|CPC	2.0	6.2	**8.2**
2c	DSL|CDWpL|QL *|CPL	2.0	7.4	**9.4**
3	DSC|QC *|CPC	2.0	3.5	**5.5**
3	DSL|QL *|CPL	2.0	5.1	**7.1**

* Locations where crushing takes place.

**Table 7:** Comparison of the distance (in km) between the first CDW plant and the alternative one.

	First CDW Plant	Alternative CDW Plant
**Demolition site—CDW plant**	25.4	13.7
**CDW plant—quarry**	35.5	10.1
**CDW plant—concrete plant**	76.9	42.3

## Data Availability

The data presented in this study are available on request from the corresponding author.

## References

[1] EU Directive 2008/98/EC of the European Parliament and the Council of 19 November 2008 on Waste and Repealing Certain. https://www.legislation.gov.uk/eudr/2008/98/chapter/I.

[2] Xiao J., Li W., Fan Y., Huang X. (2012). An overview of study on recycled aggregate concrete in China (1996–2011). Constr. Build. Mater..

[3] Pacheco J., de Brito J., Ferreira J., Soares D. (2015). Destructive horizontal load tests of full-scale recycled aggregate concrete structures. ACI Struct. J..

[4] Tošić N., Torrenti J.M., Sedran T., Ignjatović I. (2021). Toward a codified design of recycled aggregate concrete structures: Background for the new Fib Model Code 2020 and Eurocode 2. Struct. Concr..

[5] prEN1992(2020). prEN 1992-1-1 D6 Working file (2020-10-05 Rev. 7), CEN/TC-250/SC-2.

[6] Braga A.M., Silvestre J.D., de Brito J. (2017). Compared environmental and economic impact from cradle to gate of concrete with natural and recycled coarse aggregates. J. Clean. Prod.

[7] Tošić N., Marinković S., Dašić T., Stanić M. (2015). Multicriteria optimization of natural and recycled aggregate concrete for structural use. J. Clean. Prod..

[8] Hossain M.U., Poon C.S., Lo I.M.C., Cheng J.C.P. (2016). Comparative environmental evaluation of aggregate production from recycled waste materials and virgin sources by LCA. Resour. Conserv. Recycl..

[9] Simion I.M., Fortuna M.E., Bonoli A., Gavrilescu M. (2013). Comparing environmental impacts of natural inert and recycled construction and demolition waste processing using LCA. J. Environ. Eng. Landsc. Manag..

[10] Rosado L.P., Vitale P., Penteado C.S.G., Arena U. (2017). Life cycle assessment of natural and mixed recycled aggregate production in Brazil. J. Clean. Prod..

[11] Kurda R., Silvestre J.D., de Brito J. (2018). Life cycle assessment of concrete made with high volume of recycled concrete aggregates and fly ash. Resources. Conserv. Recycl..

[12] Marinković S., Radonjanin V., Malešev M., Ignjatović (2010). Comparative environmental assessment of natural and recycled aggregate concrete. Waste Manag..

[13] Fraj A.B., Idir R. (2017). Concrete based on recycled aggregates—Recycling and environmental analysis: A case study of Paris’ region. Constr. Build. Mater..

[14] Park W.J., Kim T., Roh S., Kim R. (2019). Analysis of life cycle environmental impact of recycled aggregate. Appl. Sci..

[15] Estanqueiro B., Silvestre J.D., de Brito J., Pinheiro M.D. (2018). Environmental life cycle assessment of coarse natural and recycles aggregates for concrete. Eur. J. Environ. Civ. Eng..

[16] Jullien A., Proust C., Martaud T., Rayssac E., Ropert C. (2012). Variability in the environmental impacts of aggregate production. Resour. Conserv. Recycl..

[17] Göswein V., Gonçalves A.B., Silvestre J.D., Freire F., Habert G., Kurda R. (2018). Transportation matters—Does it?. GIS-based comparative environmental assessment of concrete mixes with cement, fly ashes, natural and recycled aggregates. Resour. Conserv. Recycl..

[18] Colangelo F., Petrillo A., Cioffi R., Borrelli C., Forcina A. (2018). Life cycle assessment of recycled concretes: A case study in southern Italy. Sci. Total. Environ..

[19] Pradhan S., Tiwari B.R., Kumar S., Barai S. (2019). Comparative LCA of recycled and natural aggregate concrete using Particle Packing Method and conventional method of design mix. J. Clean. Prod..

[20] Silva R.V., de Brito J., Dhir R.K. (2014). Properties and composition of recycled aggregates from construction and demolition waste suitable for concrete production. Constr. Build. Mater..

[21] Pepe M., Grabois T.M., Silva M.A., Tavares L.M., Romildo D.T.F. (2018). Mechanical behaviour of coarse lightweight, recycled and natural aggregates for concrete. Proc. Inst. Civ. Eng. Constr. Mater..

[22] Bravo M., de Brito J., Pontes J., Evangelista L. (2017). Shrinkage and creep performance of concrete with recycled aggregates from CDW plants. Mag. Concr. Res..

[23] Visintin P., Xie T., Bennett B. (2020). A large-scale life-cycle assessment of recycled aggregate concrete: The influence of functional unit, emissions allocation and carbon dioxide uptake. J. Clean. Prod..

[24] Thomas C., Setién J., Polanco J.A., Alaejos V., de Juan M.S. (2013). Durability of recycled aggregate concrete. Constr. Build. Mater..

[25] Pacheco J., de Brito J., Ferreira J., Soares D. (2015). Flexural load tests of full-scale recycled aggregates concrete structures. Constr. Build. Mater..

[26] Tošić N., Kurama Y. (2020). Parametric numerical study on service-load deflections of reinforced recycled aggregate concrete slabs and beams based on fib Model Code 2010. Struct. Concr..

[27] Kurda R., Silvestre J.D., de Brito J. (2018). Toxicity and environmental and economic performance of fly ash and recycled concrete aggregates use in concrete: A review. Heliyon.

[28] Faleschini F., Zanini M.A., Pellegrino C., Pasinato S. (2016). Sustainable management and supply of natural and recycled aggregates in a medium-size integrated plant. Waste Manag..

[29] Josa I., Tošić N., Marinković S., de la Fuente A., Aguado A. (2021). Sustainability-oriented multi-criteria analysis of different continuous flight auger piles. Sustainability.

[30] Braga A.M. (2015). Compared environmental impact of the life cycle of concrete with natural and recycled coarse aggregates (in Portuguese). Master’s Thesis.

[31] Kurda R. (2017). Sustainable development of cement-based materials: Application to recycled aggregates concrete. Ph.D. Thesis.

[32] Della Transport prices Europe. https://della.eu/price/local/.

